# Reactivity-based identification of oxygen containing functional groups of chemicals applied as potential classifier in non-target analysis

**DOI:** 10.1038/s41598-023-50240-y

**Published:** 2023-12-20

**Authors:** Milena Latz, Alexander Böhme, Nadin Ulrich

**Affiliations:** 1 Department of Ecological Chemistry, Helmholtz Centre for Environmental Research - UFZ, 04318 Leipzig, Germany; 2 Faculty of Chemistry and Mineralogy, Leipzig University, 04103 Leipzig, Germany

**Keywords:** Chemistry, Analytical chemistry

## Abstract

In this work, we developed a reactivity-based strategy to identify functional groups of unknown analytes, which can be applied as classifier in non-target analysis with gas chromatography. The aim of this strategy is to reduce the number of potential candidate structures generated for a molecular formula determined by high resolution mass spectrometry. We selected an example of 18 isomers with the molecular formula C_12_H_10_O_2_ to test the performance of different derivatization reagents, whereas our aim was to select mild and fast reaction conditions. Based on the results for the isomers, we developed a four-step workflow for the identification of functional groups containing oxygen.

## Introduction

In today’s globalized world, industrial and agricultural chemicals, pharmaceuticals, and personal care products are omnipresent in our daily life, exposing humans and the environment to a cocktail of chemicals^1^. With over 235,000 chemicals^2^ being produced globally to this day, public concerns and scientific findings support the need for sufficient protection and regulation against chemical exposure. Resulting of the increased production, use, and disposal of chemical products and the accompanied adverse impact, the term “anthropogenic chemical pollution” was shaped. The EU chemicals strategy for sustainability (CSS) for safer and ecological handling of chemicals was introduced to steer development in another direction^3^. Apart from the sheer amount of chemicals emitted, another significant difficulty arises: the recent chemical industry can be seen as a fluid system with new substances being developed constantly, leading to major regulatory difficulties for these chemicals. Implementing substance-specific policies is a tedious and lengthy process overrun by the constant production of new substances.

Moreover, environmental degradation and biotransformation of chemicals lead to the formation of new, likewise ill-regulated transformation products with unknown effects on the environment and society^2^. Though, monitoring and analyzing chemical compounds affecting the environment remains challenging. One main reason is the complexity of environmental samples, containing several thousands of detectable compounds^4,5^. Identification and quantification of the unknowns can therefore be seen as the big challenge in areas like toxicology, forensics, and environmental sciences.

Non-target screening (NTS) has been implemented as a new, unbiased approach to tackle the necessity of including as many compounds as possible in environmental analysis and toxicology. Depending on the type of sample and the sample preparation procedure, the instrument is selected first (GC-EI-MS, GC-HRMS or LC-HRMS), reducing the number of chemicals, which can be detected in the following workflow. The corresponding ion source used in the analytical instrument determines the number of chemical structures which can be detected, as well. All remaining compounds can subsequently be analyzed, maximizing the amount of extracted information from the unknowns^6,7^. To this day, data processing is still the main hurdle of NTS, making its suitable applicability highly dependent on respective follow-up strategies, including prioritizing, machine learning models, or chemometrics combined in routine workflows^5,6,8–10^.

Identifying a specific chemical underlying a particular peak in the NTS chromatogram can be extremely tedious^11,12^. First, a chemical’s molecular formula needs to be determined by the accurate mass resulting from a high-resolution mass spectrum. Second, potential candidate structures can be generated for this molecular formula using algorithms or databases such as Pubchem^13^. Finally, classifiers need to be applied as filtering tools to reduce the number of possible candidates^14^. Here, different strategies have been employed in the past to reduce the number of tentative candidates and to be able to distinguish between structurally similar compounds^15^. Examples include library matches^9,10^, ionization behavior^14^, MS/MS fragmentation patterns^14,16–18^, retention behaviour^14,19–21^, partitioning properties^22^, or boiling points^22^ of analytes. In comparison to LC-HRMS, mass spectra attained from GC-EI-(HR)MS measurements are more reproducible according to the ionization process of EI (at the standardized 70 eV). EI spectra databases such as NIST can be applied for EI spectral matching. Additionally, retention indices (for example Kovats retention index) can be used to compare the retention behavior of candidate structures^23^. Data analysis can be performed automatically using programs like AMDIS^24^.

A different approach to reduce the number of potential candidates might target functional groups of candidate structures. These groups may show different reactivities with suitable derivatization agents, enabling identification based on their specific reaction behavior. Here, derivatization agents applied initially to increase the applicability of analytes for GC/MS analysis appear as a promising tool^25–28^. While for most compounds, additional derivatization can aid to determine their structural information, it can also help to detect compounds that were initially not detectable (e.g. carboxylic acids). Commonly found derivatizations in the literature include methylation, silylation, acylation, and alkylation^28,29^, which allow the conversion of alcohols into ethers, carboxylic acids into esters as well as aldehydes and ketones into imines^30^. Ruiz-Matute et al.^31^ have summarized a broad overview of applied derivatization methods. Other experimental studies mainly focus on one, but rarely multiple derivatization reagents for their studies. The main goal is to target one specific reactivity in the sample^32–36^.

The aim of this work was to combine multiple derivatization reagents into one method, allowing for a greater spectrum of samples due to multiple targets of reactivity. For a set of 18 structural isomers with a molecular formula of C_12_H_10_O_2_, we developed a workflow demonstrating the applicability of derivatization methods to confirm or exclude the presence of functional groups, thus reducing the number of potential candidates in NTS. Hence, this work focuses on reactivity-based derivatization of functional groups of chemicals as a strategy in NTS to reduce the number of potential candidates for a peak with a respective molecular formula. To this end, several derivatization reagents are applied to investigate their selectivity toward alcohols, carboxylic acids, ethers, esters, aldehydes, and ketones by analyzing the reaction products by GC-MSD.

## Results and discussion

**Figure 1:**
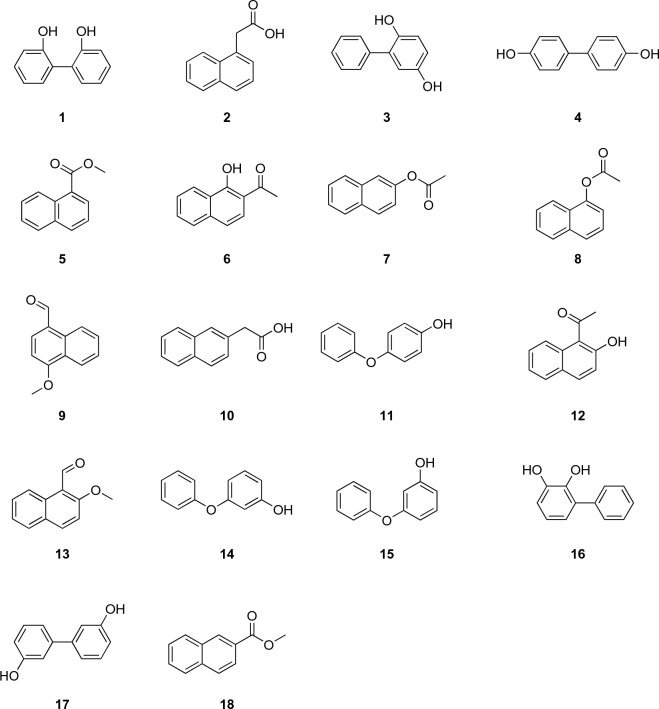
Structural formula of C_12_H_10_O_2_ isomers (MW = 186 g/mol): (1) biphenyl-2,2’-diol, (2) naphthalen-1-yl acetic acid, (3) biphenyl-2,5-diol, (4) biphenyl-4,4’-diol, (5) methyl naphthalene-1-carboxylate, (6) 1-(1-hydroxynaphthalen-2-yl) ethanone, (7) naphthalen-2-yl acetate, (8) naphthalen-1-yl acetate, (9) 4-methoxynaphthalene-1-carbaldehyde, (10) naphthalen-2-yl acetic acid, (11) 4-phenoxyphenol, (12) 1-(2-hydroxynaphthalen-1-yl) ethanone, (13) 2-methoxynaphthalene-1-carbaldehyde, (14) 3-phenoxyphenol, (15) 2-phenoxyphenol, (16) biphenyl-2,3-diol, (17) biphenyl-3,3’-diol, (18) methyl naphthalene-2 carboxylate.

We selected the molecular formula C_12_H_10_O_2_ as an example, where 279 possible isomers were found in the database Chemspider^20^. We used 18 commercially available isomers (Fig. 1, Information on suppliers given in SI1) and tested whether these chemicals were reactive towards various derivatization reagents. The derivatization reagents used here are: trimethylsulfonium hydroxide (TMSH), trifluoroacetic anhydride (TFAA), *N*,*N*-dimethylformamide dimethyl acetal (DMF-DMA), trimethylsilyldiazomethane (TMSCHN_2_), ammonium hydroxide (NH_4_OH), and tris(2,2,2-trifluoroethyl) borate (Borate), respectively. Details on the suppliers and structures of the respective reagents are given in SI2.

Based on the envisaged outcomes, our aim was to develop a workflow for O-functionalities, which aids the identification of functional groups present in the structure of unknown chemicals. By using multiple derivatization tools for different functionalities, a greater amount of compounds can be incorporated, leading to a more specific outcome for sample mixtures.

### *Reactivity of the functional groups based on the selected example of isomers with molecular formula C*_*12*_*H*_*10*_*O*_*2*_

**Figure 2:**
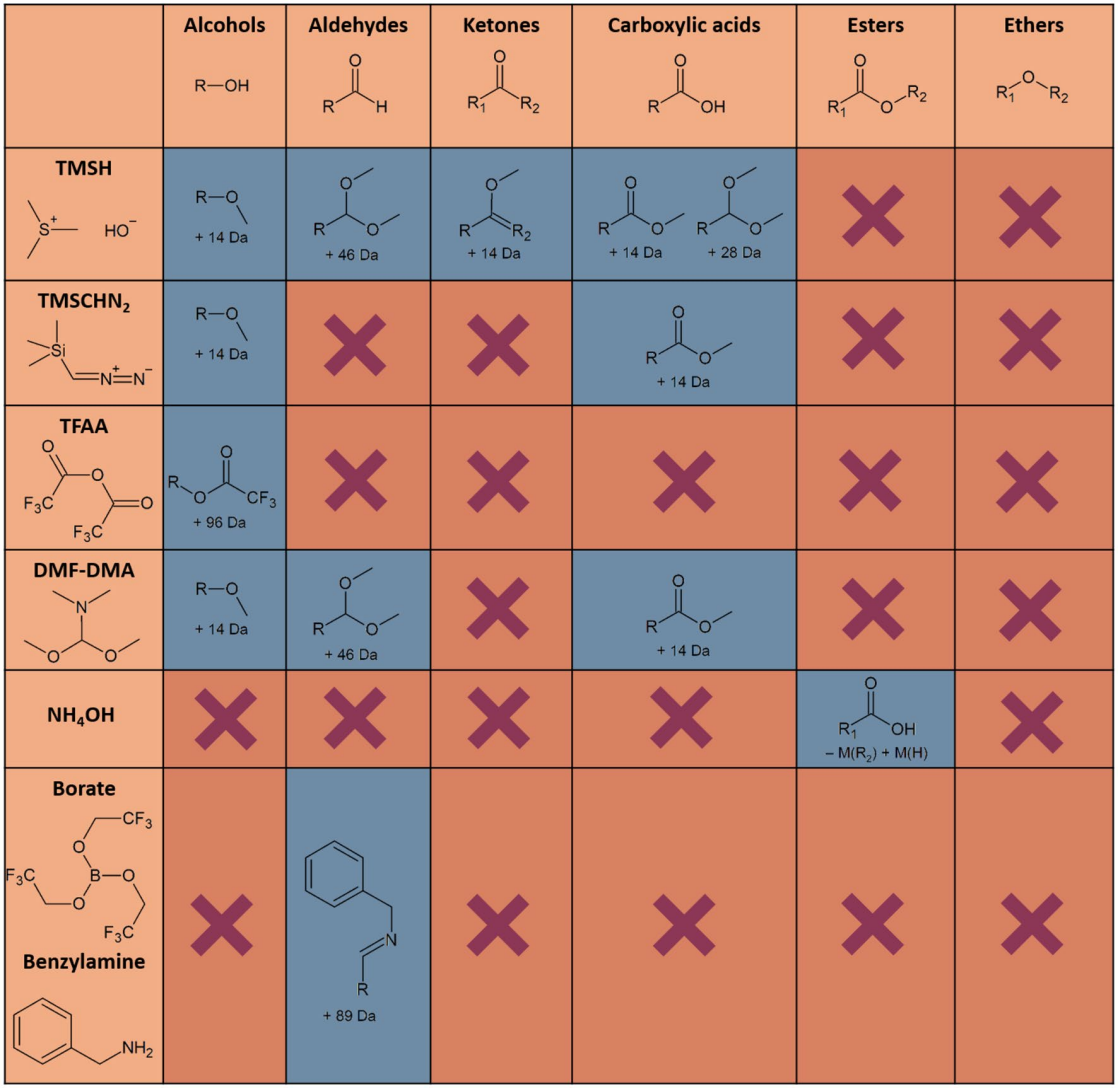
Overview on all reagents applied and results for the corresponding reactions for the example C_12_H_10_O_2_. We indicated the mass shifts observed for a successful reaction and marked (x) where we did not observe a reaction.

Figure 2 gives an overview on all reagents used in our approach and the respective target functional groups, i.e. hydroxyl group (OH), carboxylic acid (COOH), aldehyde group (CHO), carboxylic ester (COOR), keto group (COC), and ether group (ROR). We indicated the observed reaction and the mass shift in the mass spectrum or whether we did not observe a reaction. We added the details for each derivatization reaction and the individual mechanisms in the Supplementary Discussion SI3 (including Figures SI3-1 –SI3-12 on the reaction mechanisms).

By applying the derivatizing reagents proposed in Fig. 2, a sample with unknown composition can be categorized into its functional groups. For our example, all O-functionalities are covered and can be distinguished. Mainly hydroxyl groups can be identified efficiently and precisely by using TFAA. This derivatization reagent proved to be selective for hydroxyl groups under the given conditions. Another selectivity was found for aldehyde groups. When using methylating reagents such as TMSH or DMF-DMA (and partly TMSCHN_2_), distinguishing an aldehyde functionality from other groups (OH, COC, COOH) was possible as CHO showed a specific addition of + 46 Da, leading to an *m/z* of 232 as compared to the other groups for which *m/z* of 214, i.e. Δ*m* = 28 Da (2 × 14 Da) is expected. However, one should note that the methylation of the ketones (isomer 6 and 12) might be driven by keto-enol tautomerization, supported by the additional OH group and the aromatic ring (SI3 Supplementary Figure S3-4). For the reaction of alkyl ketones with TMSH a peak shift has beed observed, too. The underlying mass spectra, however, do not allow for elucidating adduct structures (see SI4 Supplementary Fig. 4–1 and 4–2). Solely ether groups were non-reactive with all reagents used and can only be categorized by excluding other possibilities. Still, exclusion has proven to be a reliable method due to the robust stability of ether bonds and their lacking potential to react with either derivatization agent.

To distinguish keto- and aldehyde groups in a better way, tris(2,2,2-trifluoroethyl) borate^37^, benzylamine, and 2,4-difluorobenzylamine have been tested as further reagents. However, the aim to distinguish aldehyde groups from other functionalities by forming an imine could not yet be reached for all isomers tested. Apart from this, a method to generate enaminones from keto-functionalities using DMF-DMA was tested^38,39^, though only the previously described methylated products could be verified in the chromatogram. This work still proved the possibility for the successful formation of aldimines for certain substances following Reeves et al.^37^.

Regarding carboxylic acids, derivatization with TMSH, TMSCHN_2_, and DMF-DMA led to methylations. Peak shape significantly improved for these chemicals after derivatization. For distinction from keto-groups, DMF-DMA proved to only methylate carboxylic acids, leading to an explicit characterization. Further options for derivatization of carboxylic acids include the direct amidation using borate esters with catalysts like amberlyst/ amberlite^40,41^ and more aggressive reaction conditions^42^. Due to increased difficulty in the reaction procedure (high reaction temperatures, long reaction times, aqueous work-up), this derivatization proved to be less suitable for this process. Even though a shift of the molecular ion peak has been observed for this derivatization, it cannot be explained by a reasonable product structure yet. It is also important to note that according to Starkov et al.^42^, the presence of a hydroxyl group leads to a significant reduction in conversion effectiveness due to an inhibition of the borate. Thus, it is difficult to use this derivatization method for unknown sample compositions.

Ester cleavage could be observed for different hydrolysis reactants and reaction conditions (basic conditions with NH_4_OH/acidic conditions, concentration of acid/base, solvent polarity, and reaction time) in absence of sterically challenging side chains^43^. Steric hindrance, however, caused the reaction to fail, not producing a cleaved product for the hydrolysis of carboxylic ester groups.

Depending on the derivatizing agent used, we also observed partial conversions of the educts with multiple functional groups. While complete derivatization of multiple functional groups was always observed for TFAA, agents such as TMSH showed partially derivatized products as well as their fully derivatized versions (e.g. isomer 1, SI5).

### Developed workflow to identify O-functionalities being present in the structure of an unknown sample

**Figure 3:**
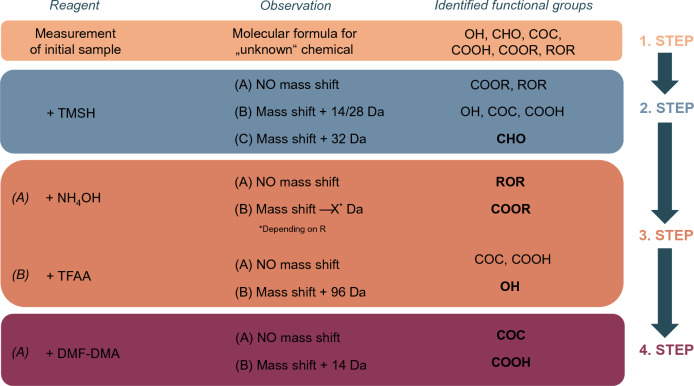
Workflow to achieve structural information for an unknown sample with known molecular weight based on the specific reactivity of the functional groups present.

Combining all derivatization methods tested throughout this work, a decision pathway for the structural analysis of samples with known empirical mass has been established. This can be especially helpful for non-target analysis approaches. In Fig. 3, the decision pathway can be seen using the example of the used empirical formula C_12_H_10_O_2_. The decision pathway allows information about distinctive structural features of the sample with easy and fast derivatizations.

Based on our findings we developed the workflow, shown in Fig. 3. In the first step of the workflow, the sample is measured in its underivatized form, providing further information like the molecular weight, if not known yet, and a reference peak in the spectrum. Subsequently, the sample should be derivatized with TMSH and analysed via GC-MSD. If no peak shift is observed, it is most likely that the sample contains either an ether- or carboxyl ester functionality. A hydrolysis reaction using NH_4_OH can be conducted to distinguish between these two functional groups. Here, ethers would again show no change in the chromatographic spectrum. In contrast, a new molecular ion peak with a smaller *m/z* value should be found for carboxyl esters, corresponding to the alcohol formed during the reaction.

In the case of a change in the spectrum after derivatization with TMSH, the sample most likely contains hydroxy-, carboxyl acid-, aldehyde-, or ketone- functionalities. Due to the occurring methylation, the new base peak should be found at higher *m/z* values with a ∆*m* of + 14 Da/ + 18 Da for OH, COOH, and COC or a ∆*m* of + 46 Da for CHO, respectively. Furthermore, methylation generally leads to a peak shift in the chromatogram to later retention times. With this information, aldehyde groups can already be distinguished from other hyphenation: methy-lated functionalities at this point, as the mass difference from the original sample is higher than for others due to a different methylation pathway (SI3 – Figure S3-3). A derivatization reaction forming an imine with borate and phenylamine can be used to confirm the presence of an aldehyde group. Here, a ∆*m* of + 189 Da compared to the original sample can be found. As this reaction did not work for all aldehydes tested, it was not included in the workflow method, though it can support the results gained so far.

To distinguish OH-, COOH-, and COC-functional groups, acylation using TFAA is highly recommended. TFAA showed specificity for hydroxyl groups by only generating a fluorinated carboxyl ester group with said functionality. Here, the molecular ion peak in the spectrum was found with a mass difference of + 192 Da for each hydroxyl group present in the analyte structure and a shift in the chromatogram to earlier retention times.

As a final step, COOH- and COC-functionalities were distinguished by reaction with DMF-DMA. Carboxylic acids were readily methylated, leading to a ∆*m* of + 14 Da or + 18 Da and a shift in the chromatographic spectrum towards later retention times. Ketones showed no reactivity with DMF-DMA. This selectivity can also help to distinguish ketones from aldehydes, as methylation was also found for CHO.

Another derivatizing reagent tested was TMSCHN_2_. It showed promising results for the methylation of OH- and COOH-functionalities and no reaction for COOR and ROR groups, and thus is recommended as a supportive reaction. Though, it is essential to note that no clear results were found for the methylation of aldehyde functionalities with this reagent.

### Validation of the workflow

**Figure 4:**
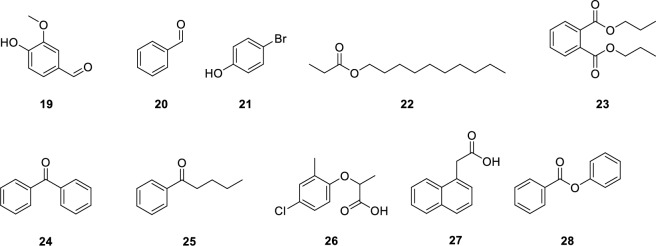
Structural formula of example mix: (19) vanillin, (20) benzaldehyde, (21) 4-bromphenol, (22) ethyldecanoate, (23) di-*n*-propylphthalate, (24) benzophenone, (25) valerophenone, (26) mecoprop, (27) naphthaleneacetic acid, (28) phenylbenzoate.

Furthermore, all 18 isomers were measured as one associated sample mix (see SI6). Due to the strong similarity of mass spectra of the isomers (especially for constitutional isomers), annotation of the respective product peaks proved to be difficult. Prediction of retention indices (using the boiling point approach or the LSER approach for example) of the corresponding derivatization products cannot be done as accurately as needed to allow for a reliable peak annotation either. Thus, we needed the individual measurements to ensure the correct peak annotation. We are aware that this is a problem when it comes to the implementation of this approach in automated workflows. Nevertheless, this issue might always occur for mixtures of isomers with similar physicochemical properties. To allow for further validation of the previously described workflow, a random sample mix containing ten compounds with different O-functionalities was chosen. Structural information on the compounds can be found in Fig. 4. Compared to previously tested isomers, more complex compounds were selected, containing up to three different functionalities. While matrix effects were omitted, a mix of all ten compounds was prepared, enabling the derivatization and subsequent analysis of all compounds in one step. The reaction procedure was done as previously described, however, analysis proved to be more complex. As a first step, the initial sample is measured without derivatization. Based on the MS spectrum of the peak found in the initial measurement, the molecular formula can be determined, and substructures can be identified using classifiers like NIST or MOLGEN-MS^44^. Subsequently, the number of potential candidate structures could be reduced using our proposed derivatization workflow (Fig. 3). In brief, all measurements were then screened separately for the derivatization agent specific products resulting from the mass shifts (*m/z* initial compound + ∆*m*). As the derivatized products are usually not included in mass spectral databases (e.g. NIST)^45^, the peak screening was done manually. To apply this strategy in automated workflows, the mass shifts need to be implemented in the respective software tools.More detailed results for the performed derivatization reactions can be found in the SI7.

Derivatization with TMSH, TFAA and DMF-DMA worked as expected and all the desired functionalities showed a reaction. For the hydrolysis on the other hand, some difficulties were obtained as only good leaving groups seemed to cleave from the ester, while for others no reactivity could be observed. Furthermore, most generated carboxylic acids from hydrolysis could not be detected via GC–MS. Therefore, formed carboxylic acids were in a second step methylated with TMSH to yield the corresponding methyl ester, which could then be observed in the chromatogram. The example mix proved the applicability of the proposed workflow.

In summary, the requirements for an application to an unknown sample seem promising. Though it is still to mention, that this method has yet only been established as a proof-of-concept. Moving forward, the pathway has therefore to be tested on a real-life sample using high-resolution instruments.

### Reactivity-based identification strategies for other functional groups

Apart from the tested functional groups, other functionalities such as thiol and amino groups could also be frequently present in the structures of chemicals present in environmental samples. Here, thiol functionalities are especially important in the fields of biotechnology and catalysis^46,47^. TMSH and DMF-DMA might probably also be reactive towards those functionalities. Therefore, methods for the derivatization of other functional groups should also be considered in the future.

According to literature, multiple methods are already known for direct amidation of different functionalities with borate. Reeves et al.^37^ proposed the formation of various aldimines/ ketimine with borate through condensation at room temperature, as well as the possibility for sulfinyl and sulfonyl aldimine or ketimine formation from sulfonamides/ sulfonamides.

In the case of primary amides being present in the sample, transamidation (100 °C, acetonitrile, 15 h) using borate esters could prove to be helpful. It is important to note that borate esters generally tolerated the presence of functional groups like alkenes, cyclopropanes, indoles, hydroxyl groups, esters, and Boc protection groups during derivatization reactions, making them extremely valuable for NTS^42^.

Another option for primary or secondary amines is the trifluorethylation using trifluoroacetic anhydride (TFAA)^48–50^ or trifluoroacetic acid (TFA)^48^. Due to catalyst-free conditions, various functionalities like alkenes, esters, nitro, nitrile, Boc protection groups, amides, azides, and aryl bromides were not targeted during this derivatization and remained structurally intact. The possibility of double methylation, as already seen for hydroxyl groups in the work above, was mentioned for these reactants. Though, the reaction with a secondary amide is supposedly far slower and rather unlikely to take place than for primary amines. Moreover, compared to hydroxyl groups, the reaction with primary amines is supposedly much slower^50^. Imine formation can also be achieved using aldehydes like pentafluorobenzaldehyde (PFBA)^50–52^, 4-nitrobenzaldehyde (NBA)^52^, or *para*-chlorobenzaldehyde (PCB)^49,53^. PFBA was found to be a quick and quantitative reaction agent (90% yield) when using gas phase derivatization^51^. To summarize, options for the derivatization of amines are grand and could prove to be an essential addition to the proposed approach.

Fewer derivatization reactions are found in the literature for thiol (SH) functionalities. One method, by Thiry et al.^47^ focuses on using *N*-ethylmaleimide (EMI) as a labeling option by forming a stable thioether link. After XPS analysis, the method proved to be quantitative, though not fully selective, as EMI also showed reactions with free hydroxyl groups present. A technique using *ortho*-phthalaldehyde as a derivatization agent for sulfur dioxide (SO_2_) by producing a fluorescent compound was recently employed by Mu et al.^54^.

## Conclusions

This work aimed to highlight another possible strategy to minimize the number of potential suspects for one molecular formula based on the reactivity of their-functional groups. For this, different derivatization reagents for O-functionalities were tested. A workflow method has been implemented for samples of known molecular weight but unknown molecular structure. The workflow can further be automatized using a sample preparation robot to reduce the manual workload. The proposed method has been designed as a proof-of-concept and could prove to be a helpful tool for NTS in the future. To demonstrate the proposed methods’ applicability, method validation and application to real-life samples including additional matrix-effects should be evaluated. Of course, the analysis should be conducted using high-resolution mass spectrometry instead of using single quadrupole mass spectrometry, as done here for demonstration purposes. In addition, retention time or retention index predictions can also be applied to the reaction products. However, one should note that retention time shifts might not be comparable and not occur as patterns for different isomers as hydrogen-bond donor strength (in most cases higher for the reaction educt) as well as van der Waals interactions (in most cases higher for the reaction product) do impact the retention time on DB5 columns and lead to higher retention times^20^. Thus, the overall shift in retention time or the corresponding retention index might be compensated, and the reaction product can even elute at lower retention times. We included the details on the retention indices of the isomers and their corresponding derivatization products in SI8.

## Material and methods

### Chemicals and stock solutions

18 isomers with an empirical formula of C_12_H_10_O_2_ and a molecular weight of 186.21 g/mol were used for this work. As a proof-of-concept, an example mix containing vanillin, benzaldehyde, 4-bromophenol, ethyldecanoate, di-*n*-propylphthalate, benzophenone, valerophenone, mecoprop, naphthaleneacetic acid, and phenylbenzoate was employed. CAS numbers and details on the suppliers can be found in SI1 and SI5. Structures of the isomers are given in Fig. 1 as well as of the example mix given in Fig. 4. Methanol (LC–MS grade) was purchased from Chemsolute. Formic acid (LC–MS grade) was purchased from Serva. Methyl-*tert*-butyl ether (MTBE, ultrapure, Chemsolute) was used as a solvent for all measurements. The reagents TMSH, TFAA, and DMF-DMA are purchased from Macherey–Nagel. TMSCHN_2_ is purchased from TCI. NH_4_OH and Borate are purchased from Sigma-Aldrich, and benzylamine is purchased from Merck. Details are given in SI2. For analysis, stock solutions with a concentration of 100 mg/L, 10 mg/L and 20 mg/L, respectively, in MTBE were prepared for each isomer. The stock solutions were prepared freshly from methanol stock solutions with a concentration of 10 g/L, which were stored at 4 °C over 6 months. Derivatization reagents were kept for 1 week at 4 °C.

### Derivatization of the test chemicals

Almost all reactions were performed in 2 mL glass vials, only imide formation was done in a 20 mL glass vial. The reaction products were transferred into a 2 mL glass vial with an insert (200 µL). The concentrations of the individual isomers were in the range of 10 mg/L to 100 mg/L. The concentration range was selected for demonstration of the general procedure.

For the methylation reaction with TMSH, 50 μL of 0.2 M TMSH were added to 100 μL of 20 mg/L stock solution. The mixture was subsequently analysed without additional incubation time. TMSCHN_2_ was additionally used as methylation reagent. 50 μL TMSCHN_2_ and 50 μL methanol were added to 100 μL of 100 mg/L stock solution. After an incubation time of 30 min at room temperature, 50 μL of the reaction mixture were transferred into a vial with an insert and 150 μL MTBE were added before GC-MSD analysis. The dilution was performed to reduce the amount of methanol in the sample to prevent column damage.

The third methylation reagent was the DMF-DMA mixture. Here, 50 μL DMF-DMA and 50 μL of 100 mg/L pyridine were added to 100 μL of 100 mg/L stock solution. The samples were heated at 60 °C for 15 min and 20 μL were subsequently transferred into a vial with an insert. Before GC-MSD analysis the samples were diluted with 180 μL MTBE.

Acylation was performed using TFAA. A volume of 50 μL TFAA was added to 100 μL of 100 mg/L stock solution. The samples were heated at 70 °C for 1 h. Afterward, 20 μL solution were transferred into a vial with an insert for analysis and diluted with 180 μL MTBE.

We used NH_4_OH for hydrolysis. In brief, 10 μL NH_4_OH were added to 1 mL 20 mg/L stock solution. After 15 min at room temperature, 100 μL of a 50/50 formic acid/water mixture (v/v) were added. The aqueous solution was extracted using an SPME fiber (PDMS, d_f_ 30 µm, Supelco) to perform the analysis via GC-MSD.

Borate and benzylamine were applied for imine formation. 50 μL of 100 mg/L borate, 50 μL of 100 mg/L benzylamine, and 1.8 mL MTBE were added to 100 μL of 100 mg/L stock solution. The mixture was incubated for 5 h at 80 °C. A volume of 200 μL was subsequently transferred into a glass vial with an insert for analysis.

### Chemical analysis

GC analysis was performed on a 6890 gas chromatograph (Agilent Technologies) coupled to a 5973N mass selective detector (Agilent Technologies) working in scan mode (EI source, scan parameters *m/z* 50 – 550). An Rtx-5MS column (30 m, 0.25 mm, 0.25 µm, Restek) was used for separation with helium as carrier gas and a total flow of 1.2 mL/min. The split/splitless injector (with deactivated splitless double taper liner, Agilent Technologies) was set to 230 °C in splitless mode with an injection volume of 2 µL, the transfer line was heated to 280 °C, and a temperature program was applied as follows: 50 °C (2 min), 20 °C/min to 280 °C (2 min). ChemStation (Version E.02.00, Agilent Technologies) with an implemented NIST Mass Spectral Search Program (Version 2.3) was used for acquisition and data analysis. A PDMS SPME fibre (df 30 µm, Supelco) was used to extract aqueous samples. The fiber was conditioned for 30 min at 230 °C. Extraction from the water samples was performed for 15 min. The injection was performed manually (although it could be performed automatically), the inlet parameters as well as the liner were the same compared to the liquid injection.

The isomers were measured individually and the corresponding data analysis was performed manually to identify potential derivatization products and their corresponding mass shifts. Additionally, blanks were included in the analysis. Further, each of the derivatization reactions was performed at different days to confirm the results.

### Supplementary Information

Supplementary Information.

## Data Availability

All chromatograms and mass spectra generated during this current study are available at https://doi.org/10.5281/zenodo.7429998 repository.
