# Supplementary material for: Reactivity-based identification of oxygen containing functional groups of chemicals applied as potential classifier in non-target analysis
Source: Sci Rep. 2023 Dec 20;13:22828. doi: 10.1038/s41598-023-50240-y (PMC10739825; doi:10.1038/s41598-023-50240-y)
Supplement: Supplementary file 1 — Supplementary Information. [file 41598_2023_50240_MOESM1_ESM.pdf]

Supplementary Information: Reactivity-based identification of oxygen containing functional groups of chemicals applied as potential classifier in non-target analysis

Milena Latz,[1,2] Alexander Böhme[1], Nadin Ulrich[1,*]

[1]Department of Ecological Chemistry, Helmholtz Centre for Environmental Research - UFZ, D-04318 Leipzig, Germany

[2]Faculty of Chemistry and Mineralogy, Leipzig University, D-04103 Leipzig, Germany

*Corresponding Author: Phone + 49 341 235 1818 E-mail: nadin.ulrich@ufz.de

Contents

SI1. Details on the suppliers of the isomers with empirical formula $C_{12}H_{10}O_2$. 2

SI2. Details on the derivatization reagents and the general mechanisms for derivatization. 3

SI3. Supplementary discussion - a mechanistic discussion of reactions with the selected derivatization reagents. 4

SI4. Additional methylation tests with alkylketones using TMSH. 15

SI5. Additional information on partly derivatized isomers. 16

SI6. Application to combined isomer mixture. 17

SI7. Validation of the workflow with a test mixture. 19

SI8. Retention indices for the selected isomers and their corresponding derivatization products. 20

Supplementary References 21

SI1. Details on the suppliers of the isomers with empirical formula $C_{12}H_{10}O_2$.

Supplementary Table S1. Isomers with empirical formula $C_{12}H_{10}O_2$ (MW = 186 g/mol).

No	Name	CAS	Supplier
**1**	Biphenyl-2,2'-diol	1806-29-7	Alfa Aesar
**2**	Naphthalen-1-yl acetic acid	86-87-3	Merck
**3**	Biphenyl-2,5-diol	1079-21-6	Alfa Aesar
**4**	Biphenyl-4,4'-diol	92-88-6	Alfa Aesar
**5**	Methyl naphthalene-1-carboxylate	2459-24-7	ABCR
**6**	1-(1-Hydroxynaphthalen-2-yl) ethanone	711-79-5	Sigma
**7**	Naphthalen-2-yl acetate	1523-11-1	MP Biochemicals
**8**	Naphthalen-1-yl acetate	830-81-9	Applichem
**9**	4-Methoxynaphthalene-1-carbaldehyde	15971-29-6	Aldrich
**10**	Naphthalen-2-yl acetic acid	581-96-4	MP Biochemicals
**11**	4-Phenoxyphenol	831-82-3	Alfa Aesar
**12**	1-(2-Hydroxynaphthalen-1-yl) ethanone	574-19-6	ABCR
**13**	2-Methoxynaphthalene-1-carbaldehyde	5392-12-1	Alfa Aesar
**14**	3-Phenoxyphenol	713-68-8	Alfa Aesar
**15**	2-Phenoxyphenol	2417-10-9	Key Organics Ltd
**16**	Biphenyl-2,3-diol	1133-63-7	Fluka
**17**	Biphenyl-3,3'-diol	612-76-0	Aldrich
**18**	Methyl naphthalene-2-carboxylate	2459-25-8	Alfa Aesar

SI2. Details on the derivatization reagents and the general mechanisms for derivatization.

Supplementary Table S2. Derivatizing reagents with their corresponding CAS number and supplier.

No.	Name	CAS	Supplier
19	**TMSH** – Trimethylsulfonium hydroxide	17287-03-5	Macherey-Nagel
20	**TMSCHN$_2$** – Trimethylsilyldiazomethane	18107-18-1	TCI
21	**TFAA** – Trifluoroacetic anhydride	407-25-0	Macherey-Nagel
22	**DMF-DMA** – N, N-Dimethylformamide dimethyl acetal	4637-24-5	Macherey-Nagel
23	**NH$_4$OH** – Ammonium hydroxide	1336-21-6	Sigma Aldrich
24	**Borate** – Tris(2,2,2-trifluoroethyl) borate	659-18-7	Aldrich
25	**H$_2$NBn** – Benzylamine	100-46-9	Merck

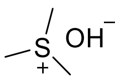

19

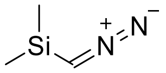

20

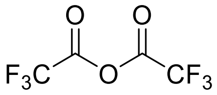

21

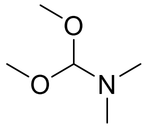

22

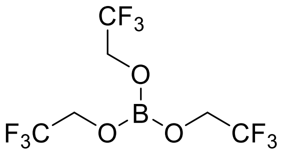

24

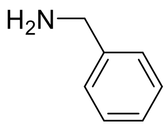

25

Supplementary Figure S2. Derivatizing reagents: TMSH – trimethylsulfonium hydroxide **19**; TMSCHN$_2$ – Trimethylsylildiazomethane **20**; TFAA – Trifluoroacetic anhydride **21**; DMF-DMA – *N*, *N*-dimethylformamide dimethyl acetal **22**; NH$_4$OH – ammonium hydroxide **23** Borate – tris(2,2,2-trifluoroethyl) borate **24**, H$_2$NBn – benzylamine **25**.

SI3. Supplementary discussion - a mechanistic discussion of reactions with the selected derivatization reagents.

Methylation of [OH, COOH, COC, CHO] via TMSH (19)

As a derivatization reagent, TMSH **19** is used as a “methyl group donor” [1]. The reagent proved to have a simple reaction procedure at room temperature and a high effectiveness for the methylation of most functional groups (hydroxyl-, aldehyde- and keto- groups as well as carboxylic acids). Generally, the derivatization of one functional group via methylation led to an ΔM of +14 Da and a new peak at *m/z* 200 Da. Double methylation was possible when two functional groups were present in the analyzed structure, such as for biphenyls 1, 3, 4, 16, and 17. In this case, two methyl groups were added, leading to an ΔM of +28 Da and the product peak at *m/z* 214 Da.

Methylation generally occurs under strong basic conditions. For example, the hydroxyl group reaction mechanism can be found in *Supplementary Figure S3-1.* Free hydroxyl anions from the derivatizing reagent remove the acidic proton from the functional group. The produced nucleophile can form a transition state with the remaining trimethyl sulfonium cation. A thioether bond is formed by nucleophilic attack, which is subsequently cleaved to form the methylated product 31 and dimethyl sulfide as a by-product. By derivatization of the second hydroxyl group, the double methylated product **26** is formed. [1]

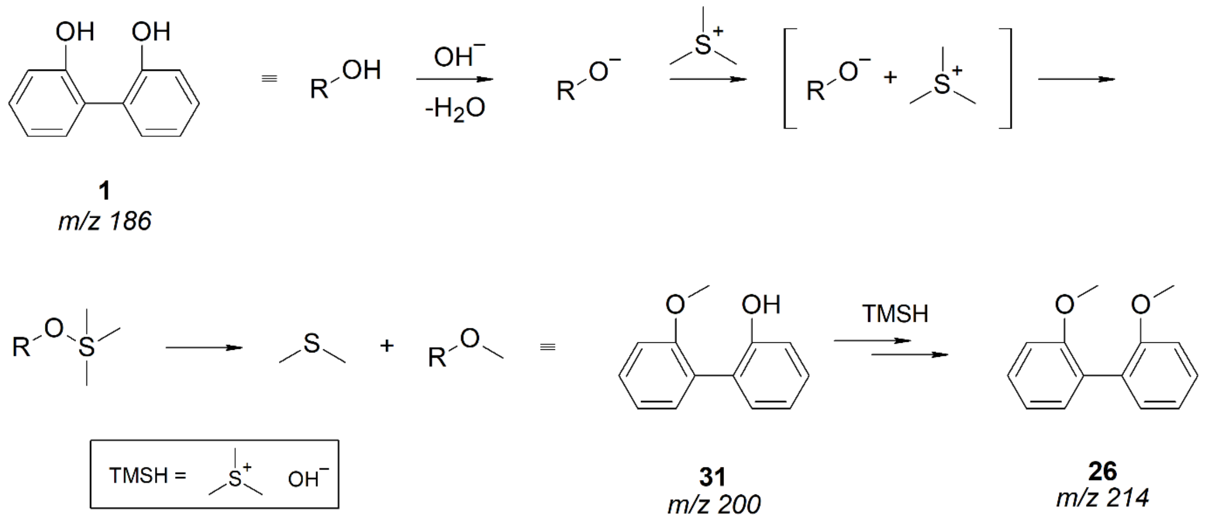

Supplementary Figure S3-1. Proposed reaction mechanism for the methylation of hydroxyl groups (OH) via TMSH **19** on the example of biphenyl-2,2’-diol **1**.

According to Gries et al. [2], the methylation of carboxylic acids via TMSH **19** (*Supplementary Figure S3-2*) follows a similar reaction mechanism as the previously explained methylation of hydroxyl groups. Again, the hydroxyl anion serves as a proton acceptor, while the produced nucleophile attacks the

trimethyl sulfonium cation. The formed carboxylic ester **28** leads to a product peak at *m/z* 200 Da. In the case of carboxylic acid **10**, a second peak at *m/z* 214 Da, representing double methylation, was even observed. A possible explanation would be a tautomerization, producing a free hydroxyl group which can then subsequently be methylated as well, leading to the product **32**. As tautomerization for carboxylic acids is generally less favored, only a fraction of the initial compound showed double-methylation, producing two peaks in the chromatographic spectrum at 10.894 min and 11.060 min, respectively. [3]

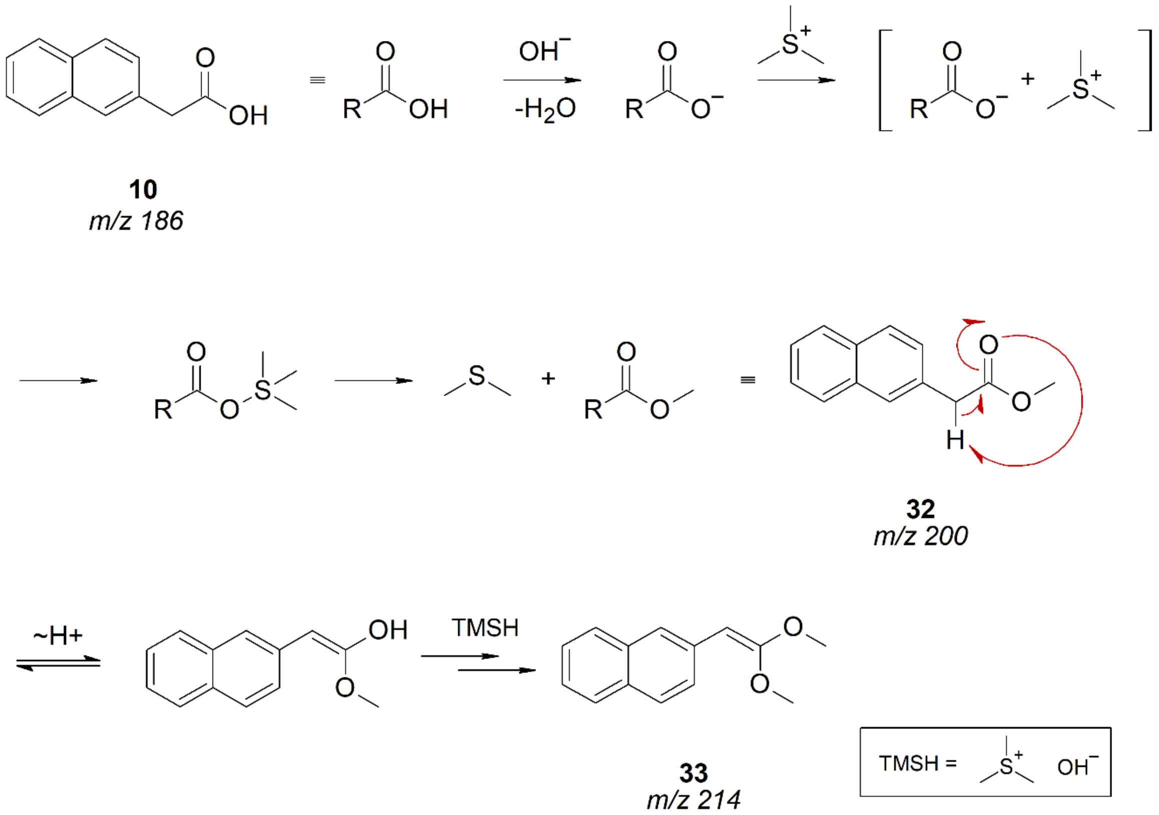

Supplementary Figure S3-2. Proposed reaction mechanism for the methylation of carboxylic acids (COOH) via TMSH **19** on the example of naphthalen-2-yl acetic acid **10**.

Interestingly, aldehyde and keto groups were also methylated in some form. For aldehyde functionalities, the product peak was measured at *m/z* 232 Da, leading to an addition of +46 Da. Here, the free hydroxyl anion can start a nucleophilic attack at the electrophilic carbon in the aldehyde functionality, leading to the presence of two hydroxyl groups, which both can be methylated by TMSH **19**, following the reaction mechanism in *Supplementary Figure S3-3.* Double methylation led to an ΔM of +46 Da and subsequently to an *m/z* of 232 Da in product **34**. The base peak at *m/z* 201 Da is possibly caused by the loss of CH_3O^+ from the methyl esters present. The differences measured between the two aldehydes **9** and **13** can be explained due to steric hindrance.

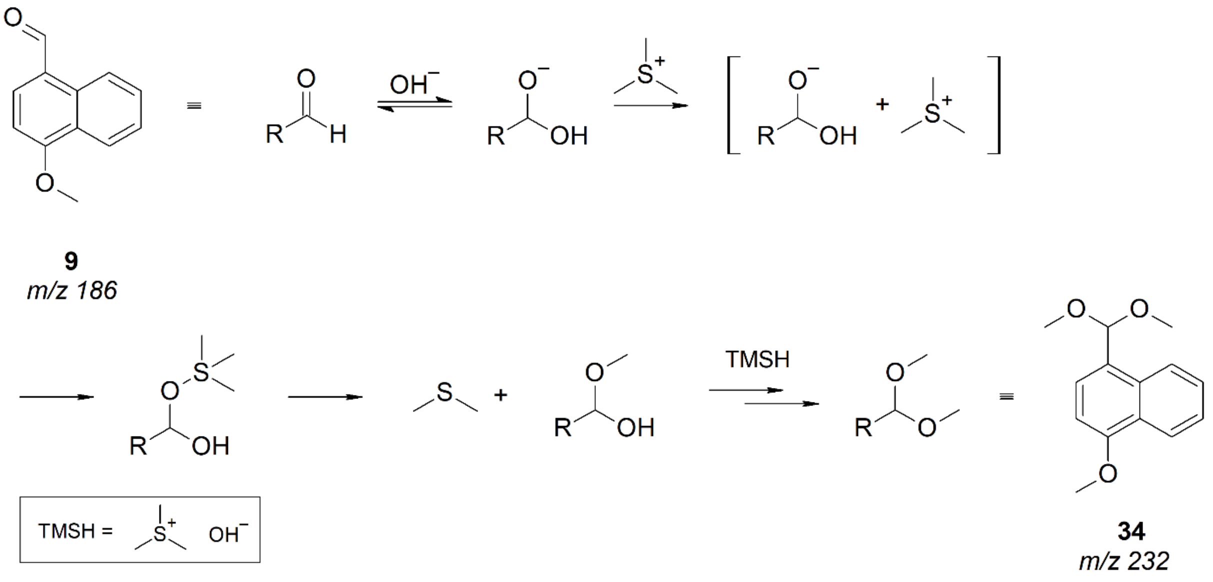

Supplementary Figure S3-3. Proposed reaction mechanism for the methylation of aldehyde groups (CHO) via TMSH **19** on the example of 4-methoxynaphtalene-1-carbaldehyde **9**.

A similar mechanism may also hold for ketones, keeping in mind that the first step, the addition of the OH^- to the carbonyl carbon, is less efficient as compared to aldehydes because of steric and electronic effects of the additional alkyl group. This lower reactivity of ketones may require higher TMSH concentration to yield sufficient turnover rates. Ketones that can be converted into corresponding enols such as substrate **6** can be methylated in analogy to alcohols (*Supplementary Figure S3-4*) and are expected to generate adducts with Δm = 14 Da (product **35**). In case of product **35,** further keto-enol tautomerization allows for a second methylation leading to product **36** with *m/z* of 200 Da.

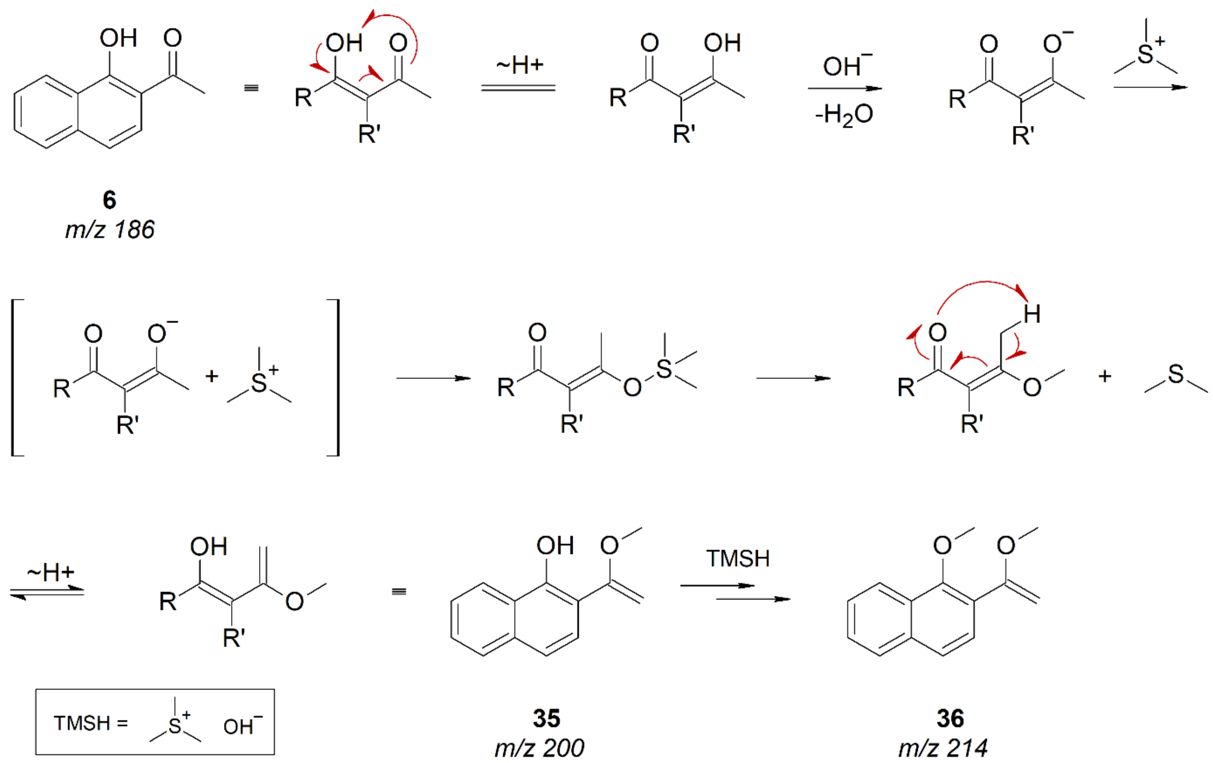

Supplementary Figure S3-4. Proposed reaction mechanism for the methylation of keto groups (COC) via TMSH **19** on the example of 1-(1-hydroxynaphthalen-2-yl) ethenone **6**.

Methylation of [OH, COOH, CHO] via $TMSCHN_2$ (20)

Like TMSH **19**, derivatization with $TMSCHN_2$ **20** was done at room temperature, where carboxylic acids and hydroxyl groups were easily methylated. The procedure is straightforward and quick, though adding MeOH is necessary. Here also, methylation leads to an ΔM of 14 Da and an *m/z* of 200 Da for single methylations and to an ΔM of 28 Da and an associated peak at *m/z* 214 Da for double methylated products.

The methylation of carboxylic acids by $TMSCHN_2$ follows the reaction mechanism depicted in *Supplementary Figure S3-5*.[5] In the first step, a proton is transferred from the carboxy group of the analyte to $TMSCHN_2$, which is now ready to react further with methanol to form TMS-OCH_3 and diazomethane. The latter is the alkylating agent tranfering its methyl group to the deprotonated analyte.[5]

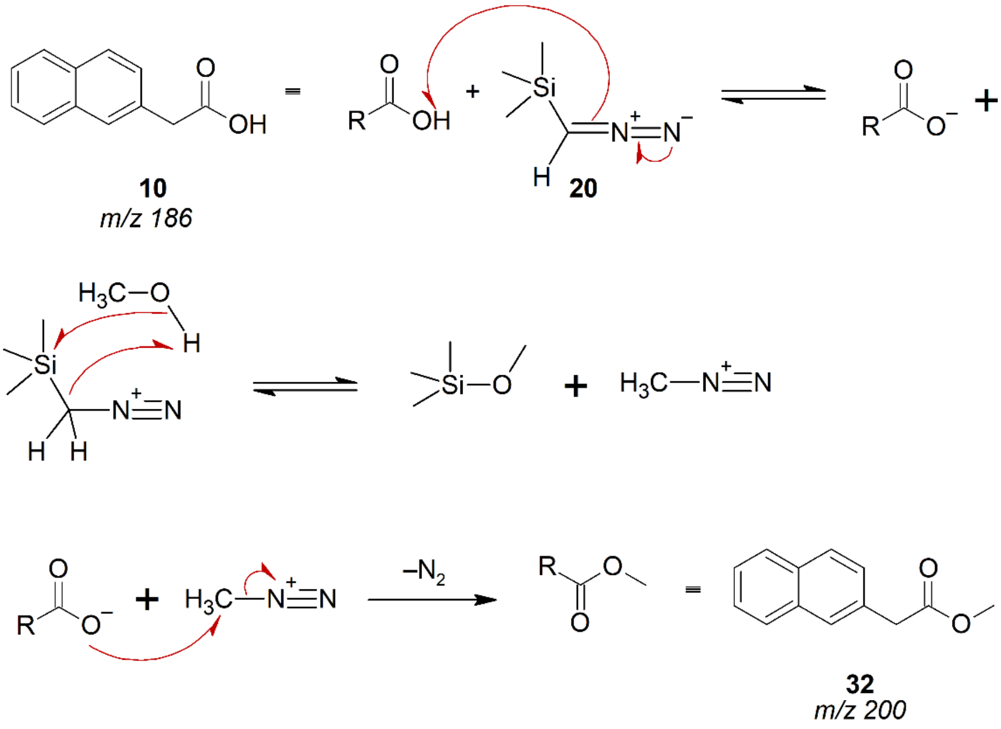

Supplementary Figure S3-5. Proposed reaction mechanism for the methylation of carboxylic acids (COOH) via $TMSCHN_2$ **20** on the example of naphthalen-2-yl-acetic acid **10**.

The methylation of hydroxyl groups follows a similar mechanism as for carboxylic acids and can be found in *Supplementary Figure S3-6.* Compared to TMSH **19**, $TMSCHN_2$ **20** is less specific for aldehyde functionalities. Dependent on the steric hindrance, only aldehyde **13** was double-methylated, while compound **9** showed no derivatization. Keto groups were not methylated at all.

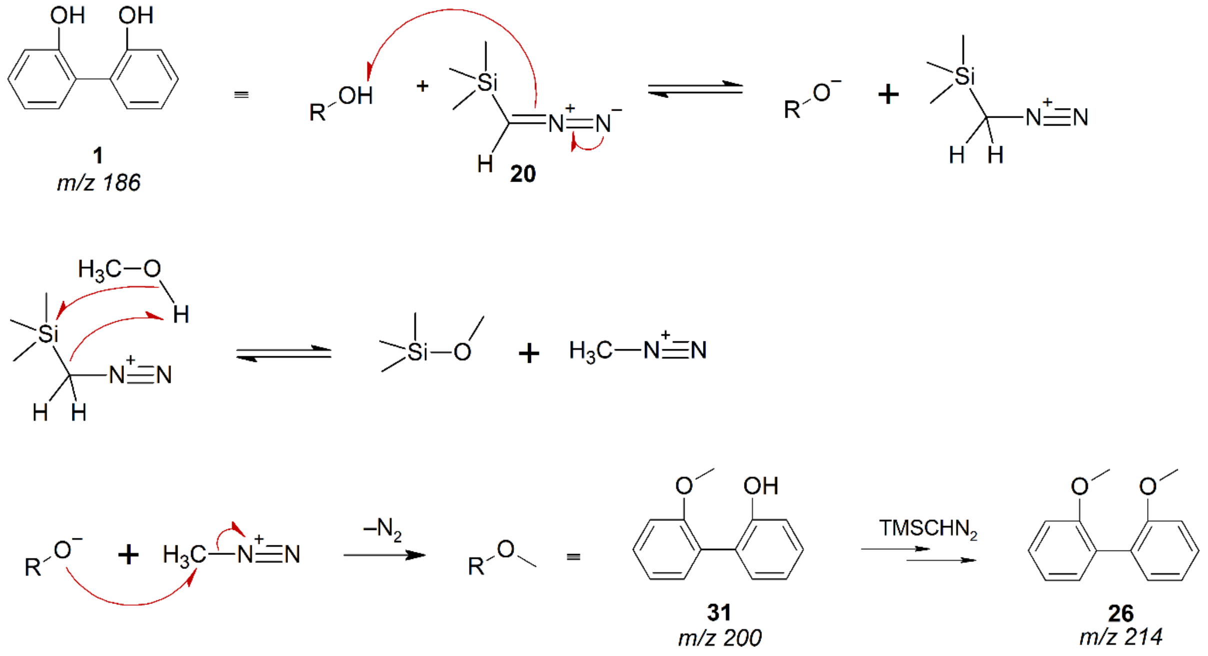

Supplementary Figure S3-6. Proposed reaction mechanism for the methylation of hydroxyl groups (OH) via TMSCHN₂ **20** on the example of biphenyl-2,2’-diol **1**.

Acylation of [OH] via TFAA (21)

To selectively distinguish alcohols from other functional groups, TFAA **21** can be used as a derivatizing reagent. Due to the fluorine content, TFAA **21** is more reactive than traditional anhydrides. The reaction mechanism follows a nucleophilic acyl substitution, acylating the hydroxyl group under acidic conditions. [7] Other functional groups containing a hydroxyl part, such as carboxylic acids, are not reactive enough and therefore do not show acylated products.

The reaction mechanism can be found in *Supplementary Figure S3-7.* First, the substrate starts a nucleophilic attack at the electrophilic carbon of the reagent, subsequently leading to a hydrogen abstraction forming the tetragonal intermediate. After rearrangement, the fluorinated carboxylate is removed as a leaving group, yielding the esterified product **37**. The produced carboxylate is lastly protonated to form trifluoroacetic acid (TFA) as a by-product.

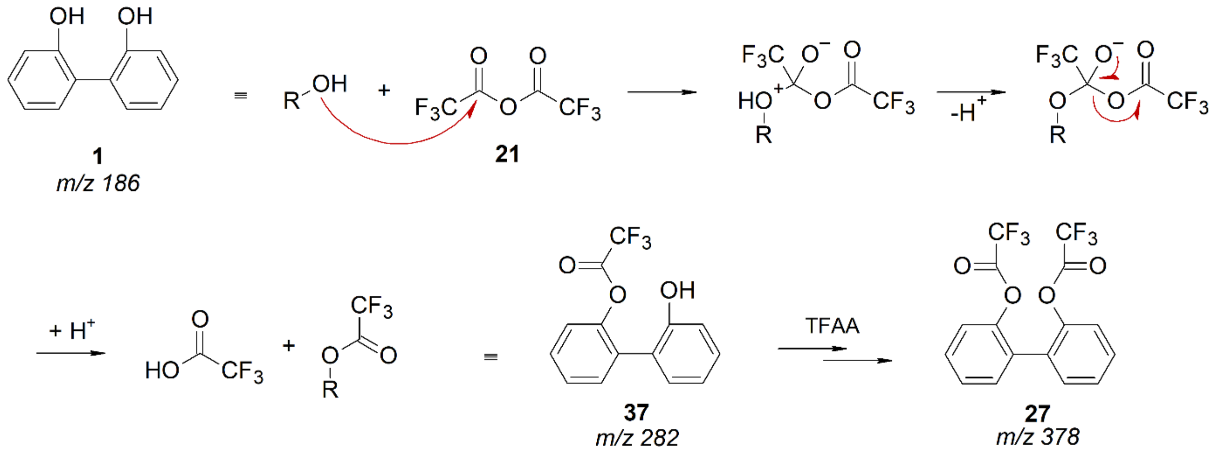

Supplementary Figure S3-7. Proposed reaction mechanism for the acylation of hydroxyl groups (OH) via TFAA **21** on the example of biphenyl-2,2'-diol **1**.

Methylation of [COOH, OH, CHO] via DMF-DMA (22)

As a strong methylating agent, DMF-DMA **22** easily methylates all carboxylic groups except ketones under certain conditions like heating or microwave radiation [8]. It is, therefore, possible to distinguish between carboxylic acids and ketones.

As a first step, the abstraction of the acidic proton leads to the formation of a carboxyl anion. By removing MeOH as a leaving group, the protonated DMF-DMA **22** can form an enamine. This can be seen as the crucial step, as this alkoxyimmonium ion **38** can act as a strong electrophile and can, therefore, readily be attacked by the free carboxyl anion. Here, the nucleophile can attack one of two carbons leading to two different methylation pathways. When attacking directly at the enamine double bond, a formylation mechanism is proposed (a), forming an intermediate in the next step. Another possibility is an intermolecular pathway (b), where the nucleophilic attack happens directly at the ether rest without producing another intermediate. [9] As seen in *Supplementary Figure S3-8,* the final methylated product and dimethylformamide (DMF) as a by-product are formed from both pathways.

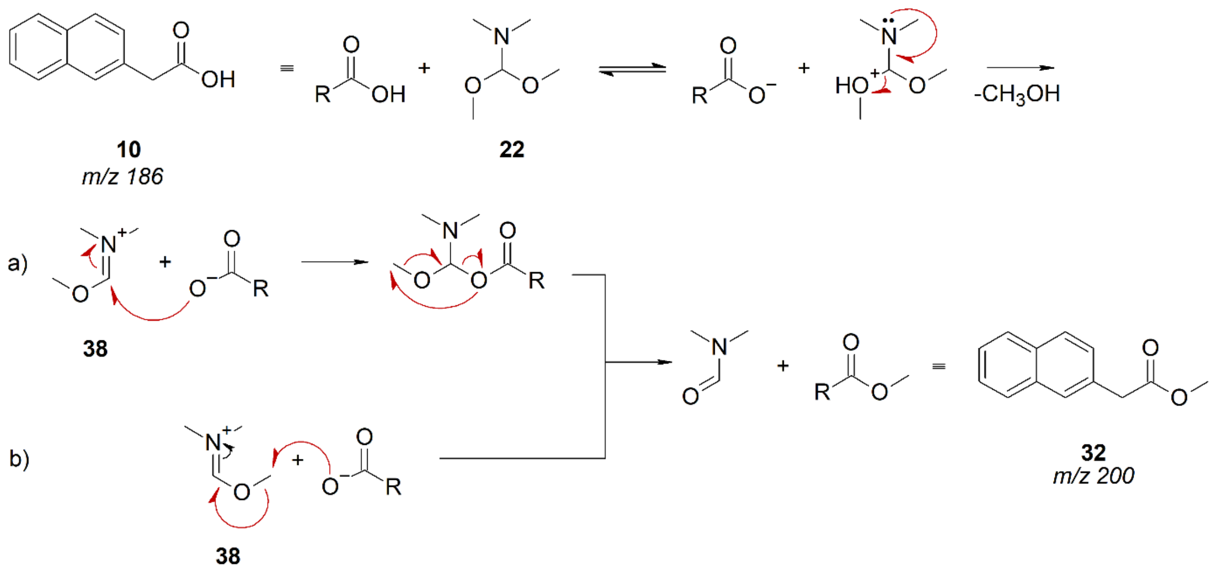

Supplementary Figure S3-8. Proposed reaction mechanism for the methylation of carboxylic acids (COOH) via DMF-DMA **22** on the example of naphthalen-2-yl acetate **10**.

The methylation of hydroxyl groups follows the same mechanism as previously explained for carboxylic acid functionalities. The only difference lays that no intermolecular pathway is possible due to the lack of another carboxylic group. The mechanism, therefore, follows the formylation pathway, as seen in *Supplementary Figure S3-9.*

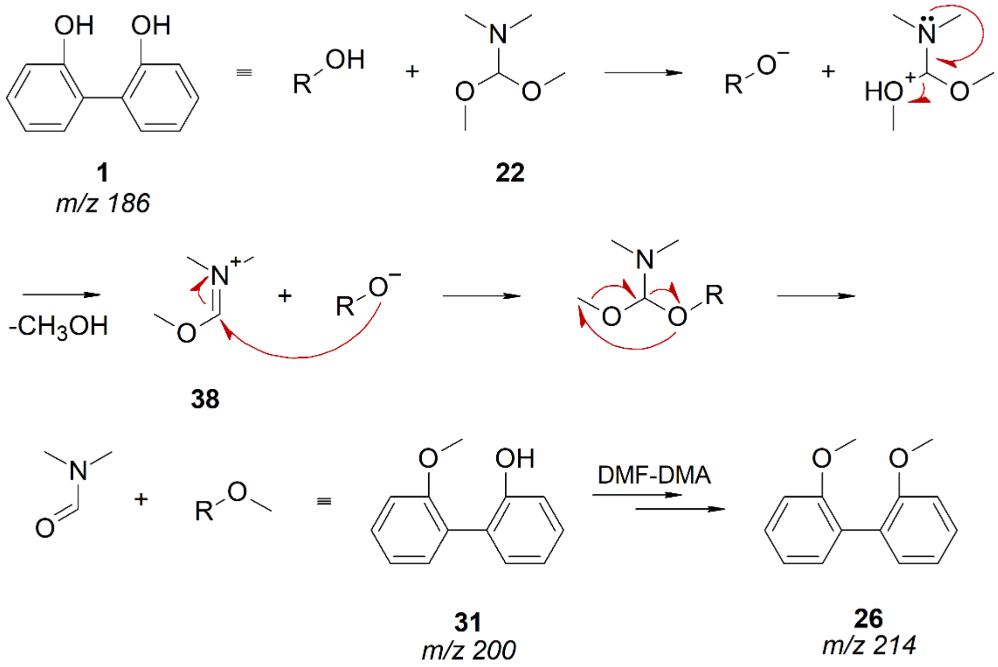

Supplementary Figure S3-9. Proposed reaction mechanism for the methylation of hydroxyl groups (OH) via DMF-DMA **22** on the example of biphenyl-2,2'-diol **1**.

For Aldehyde functionalities, double methylation leading to a mass-to-charge ratio of 232 Da has been found, as for methylation with TMSH **19**. During derivatization with DMF-DMA **22**, the produced MeOH from the formation of the alkoxyimmonion cation **38** in 1) can start a nucleophilic attack on the electrophile carbon of the aldehyde group in 2). The formed carboxylate anion can then react after a similar formylation reaction mechanism as hydroxyl and carboxyl acid functionalities. Again, the base peak at a mass-to-charge ratio of 201 Da can be based on the loss of a CH_3O^+ fragment from the methyl ester groups produced (*Supplementary Figure S3-10*).

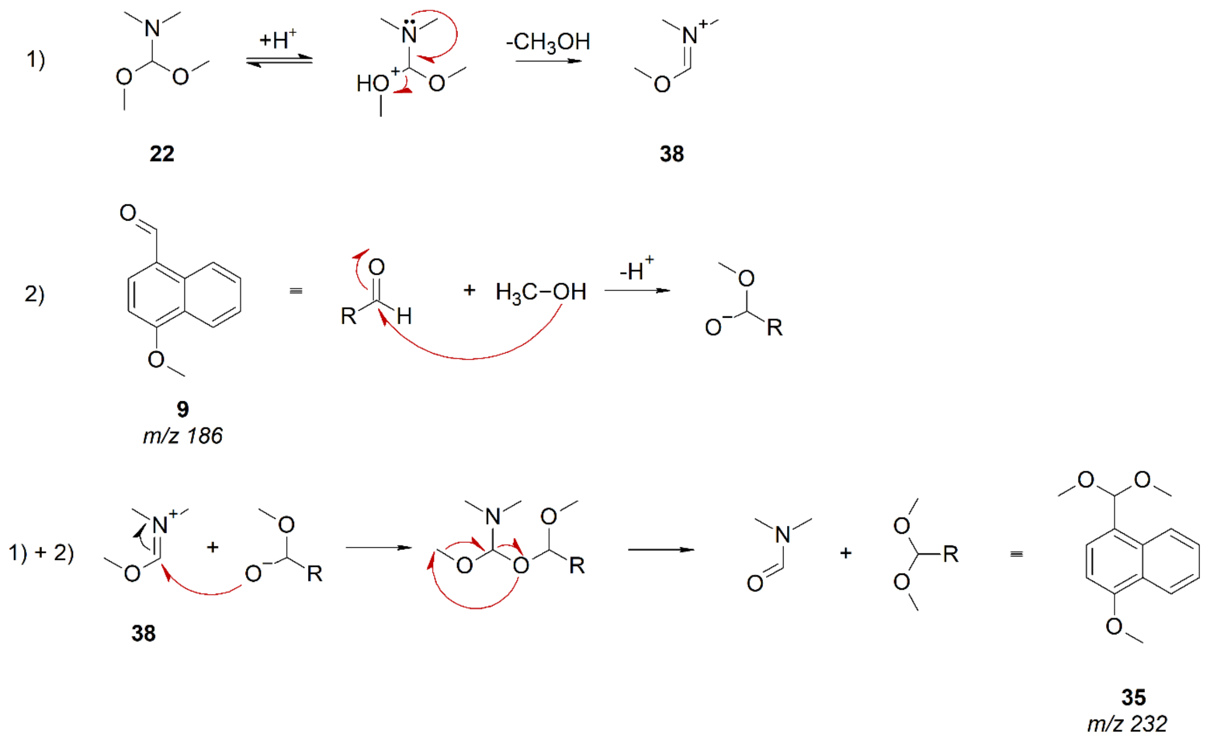

Supplementary Figure S3-10. Proposed reaction mechanism for the methylation of aldehyde groups (CHO) via DMF-DMA **22** on the example of 4-methoxynaphtalene-1-carbaldehyde **9**.

According to Chen et al. [9], methylation of aryl methyl ketones via enaminone-formation should also be possible using DMF-DMA **22** assisted by Pd/C-catalyzed hydrogenation. However, no methylation has been observed for the applied reaction conditions in this work.

Hydrolysis of [COOR] via NH_4OH (23)

As a selective procedure for carboxylic ester functionalities, a simple base-catalyzed hydrolysis reaction can be used, as seen in *Supplementary Figure S3-11*. The reaction follows an S_N2 pathway, with product formation being irreversible. At first, the free hydroxyl anion can attack as a nucleophile at the electrophilic center of the carboxylic ester, producing a tetrahedral intermediate. The intermediate collapses to regain the CO double bond by releasing an alkoxide as a leaving group and forming a

carboxylic acid. In the last, irreversible step, a proton is transferred between the two species, producing an alcohol **28**, in this case, naphthol, and the carboxylate anion. Naphthol was found in GC/MS analysis at *m/z* 144 Da. [3]

Experimental procedures proved the importance of steric hindrance and, therefore, the difficulty of generalizing hydrolysis for all carboxylic ester groups in this method. It is also possible that the produced alcohol for compounds **5** and **18**, MeOH (*m/z* 32 Da), was too small to be detected via GC/MS analysis. Therefore, further improvements for this method of derivatization should be made to distinguish carboxylic acids from other functionalities.

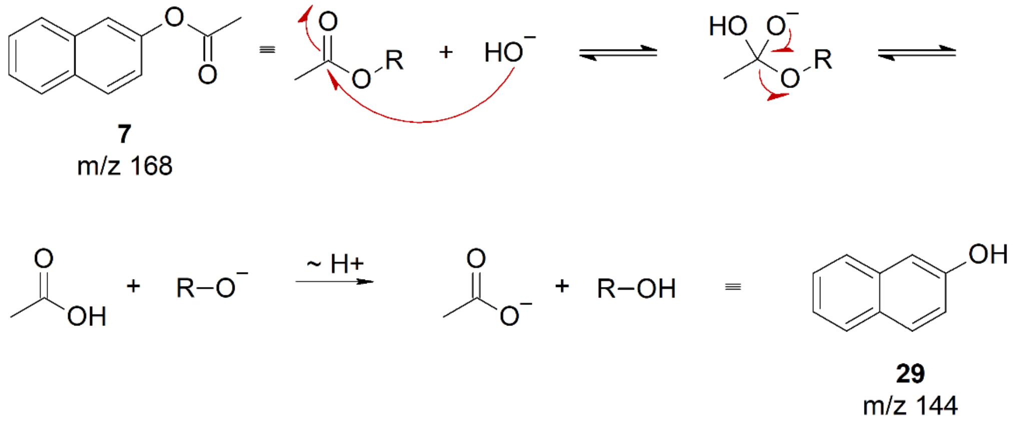

Supplementary Figure S3-11. Proposed reaction mechanism for the hydrolysis of carboxylic esters (COOR) via NH_4OH **23** on the example of naphtalen-2-yl-acetate **7**.

Imine Formation of [CHO] via borate (24) and H_2NBn (25)

The formation of an imine takes place under acidic conditions, using tris(2,2,2-trifluoroethyl) borate **24** as a proton donor, as seen in *Supplementary Figure S3-12*. The reaction mechanism follows an amide condensation. In the first step, the aldehyde is activated, possibly by the *in-situ* generation of a four-coordinated boron species. [10] Due to the fluorinated carbon rest, the borate's electrophilic properties are increased, making it an even stronger activation reagent than traditional borate esters. The electrophilic carbon can then be attacked by the introduced amine **25**. Proton abstraction leads to the subsequent formation of a hemiaminal-type structure. A good leaving group is formed through protonation, and mono-hydroxy boron species can be eliminated via condensation. The produced iminium-ion is resonance stabilized and reacts to the desired imine in the last step via proton abstraction. [3,10,11]

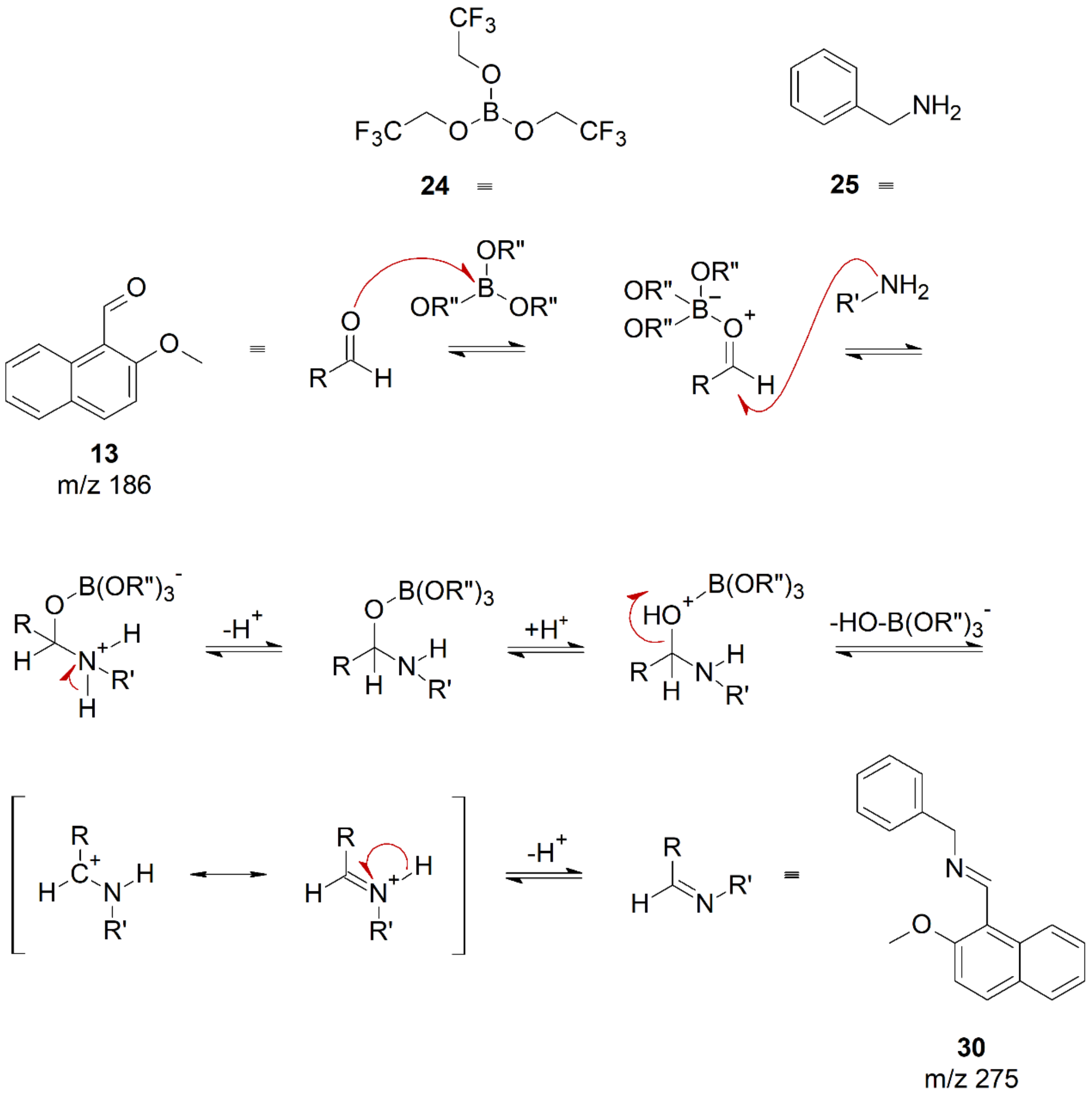

Supplementary Figure S3-12. Proposed reaction mechanism for the imine formation from aldehyde groups using borate **24** and H2NBn **25** on the example of 2-methoxynaphthalene-1-carbaldehyde **13**.

For the two aldehyde functionalities **9** and **13**, the product peak at *m/z* 275 Da was only found for **13**. The different steric environments could explain this for both compounds. Therefore, the reaction was not included in the derivatization pathway for distinguishing functional groups. Still, this reaction could be used as a confirmatory test, as it seems to work in theory but still needs further investigation. Nevertheless, the formation of imines proves to be promising as a selective derivatization reaction for aldehyde groups. Starkov et al. [10] also proposed the formation of enamines through carboxamidation of carboxylic acids and amines with borate **24**. During this work, no satisfactory results encouraging this were found. Hence, the need for conditions using higher temperatures (100 °C) and microwaves (150 W) might be interesting for future work.

SI4. Additional methylation tests with alkylketones using TMSH.

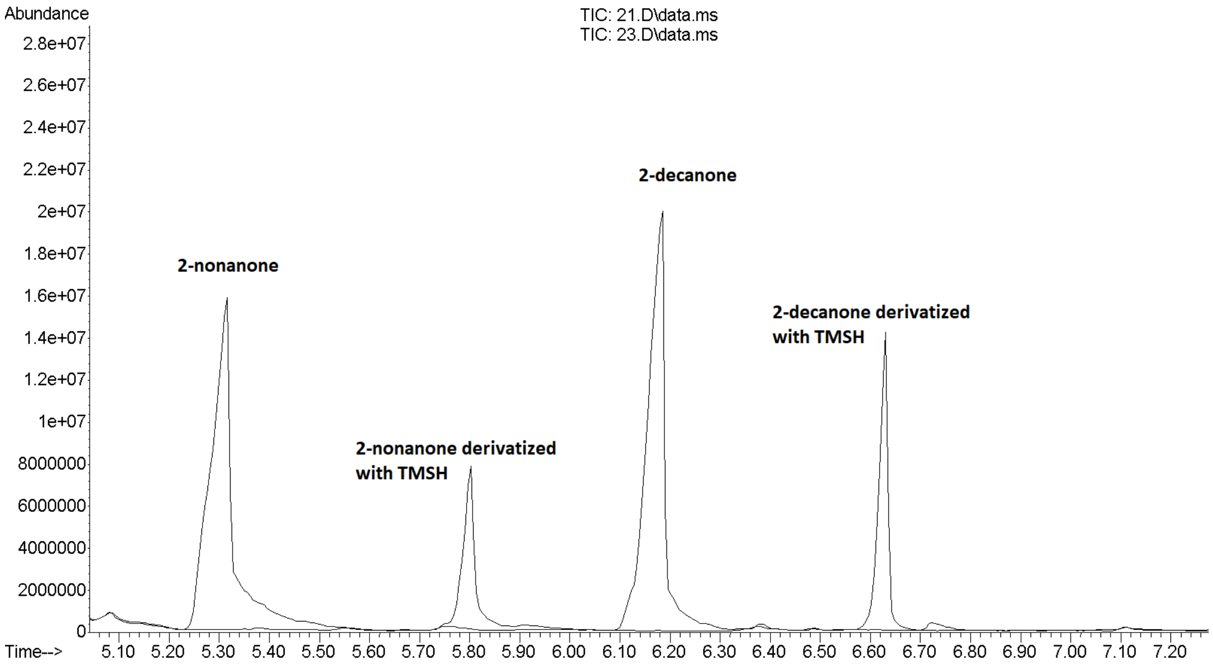

Supplementary Figure S4-1. Chromatograms of 2-nonanone and 2-decanone and their corresponding methylation products.

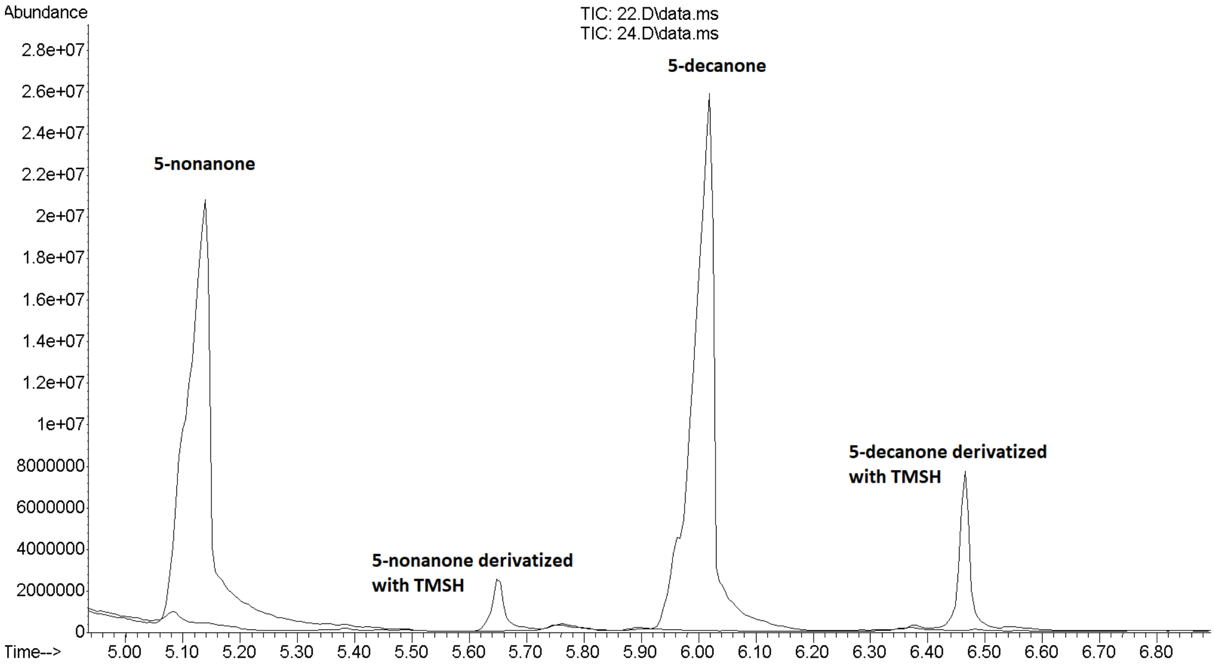

Supplementary Figure S4-2. Chromatograms of 5-nonanone and 5-decanone and their corresponding methylation products.

SI5. Additional information on partly derivatized isomers.

While we observed the fully derivatized product as the most abundant product peak in the chromatogram, indications for a partially derivatized product were also found for compounds containing more than one functional group. As an example, isomer **1**, containing two OH functionalities, produced two product peaks after derivatization with TMSH. At 10.80 min, the main derivatization product with an molecular ion of *m/z* 214 Da was found (two methyl groups included in the product structure), whereas a less intense peak at 10.72 min proved the presence of a by-product (only one methyl group included in the product structure) as well. In addition, we observed two peaks in the EIC of *m/z* 186 Da, which did not result from the initial compound **1** (RT 10.57 min), but were identified as fragments of the derivatized products. This could be confirmed by the shift in retention times.

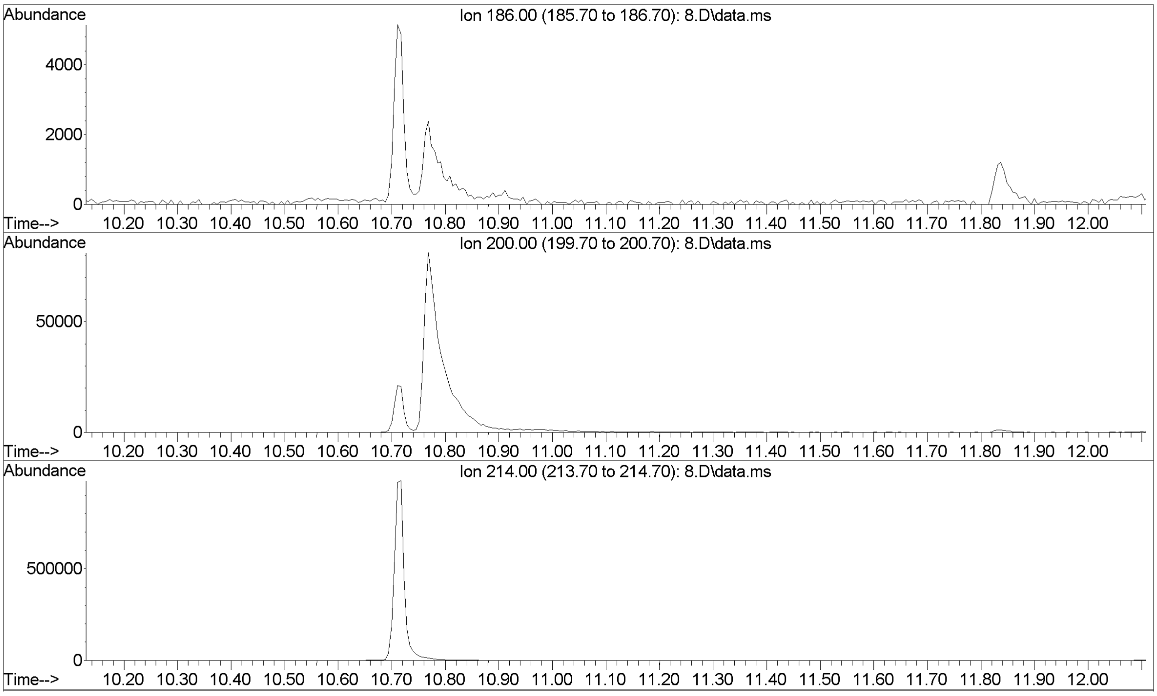

Supplementary Figure S5. EIC for the ester formation of isomer **1** with TMSH. Two OH functionalities were methylated leading to an *m/z* at 214 Da and only one OH functionality was methylated leading to an *m/z* at 200 Da.

SI6. Application to combined isomer mixture.

Additionally, we performed our workflow for one sample containing a mixture of all 18 isomers. We included the extracted ion chromatograms of the initial mixture without derivatization and the TFAA derivatization products below. The results of all other derivatization products are included in the corresponding Zenodo repository. As observed in *Suplementary Figure S6-2*, peak annotation of the received derivatization products in the combined chromatogram is problematic due to similar mass spectra of constitutional isomers and the inability to predict observed retention shifts accurately.

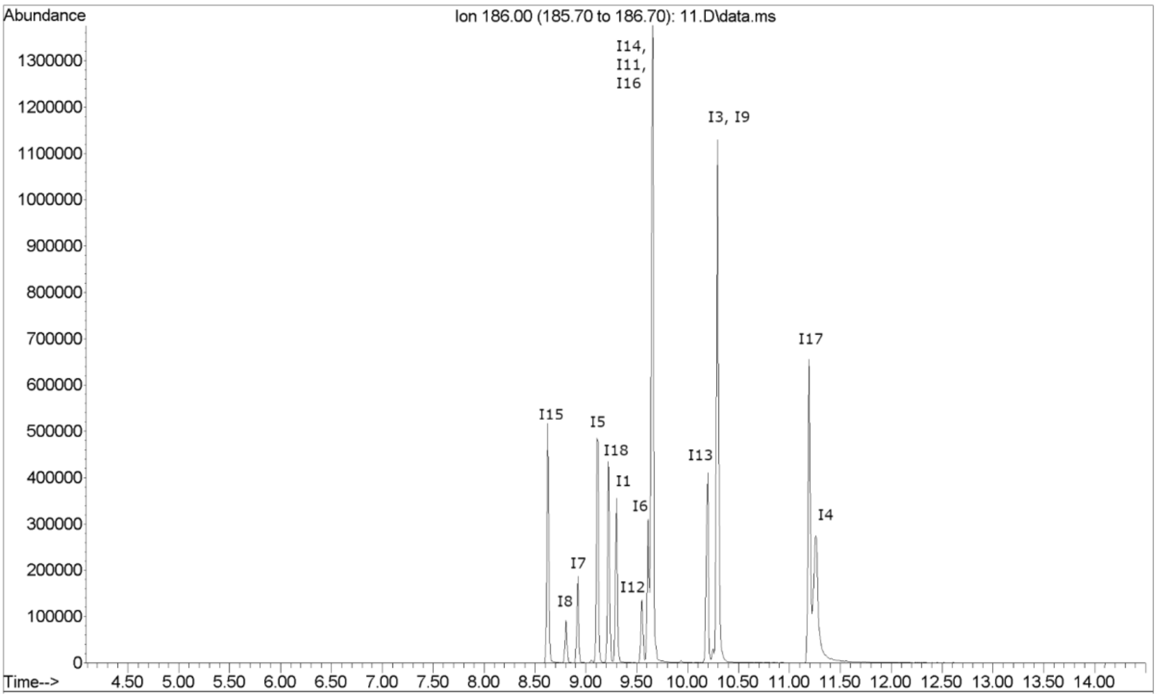

Supplementary Figure S6-1. Extracted ion chromatogram at *m/z* 186 Da of the isomer mixture including the corresponding peak annotation.

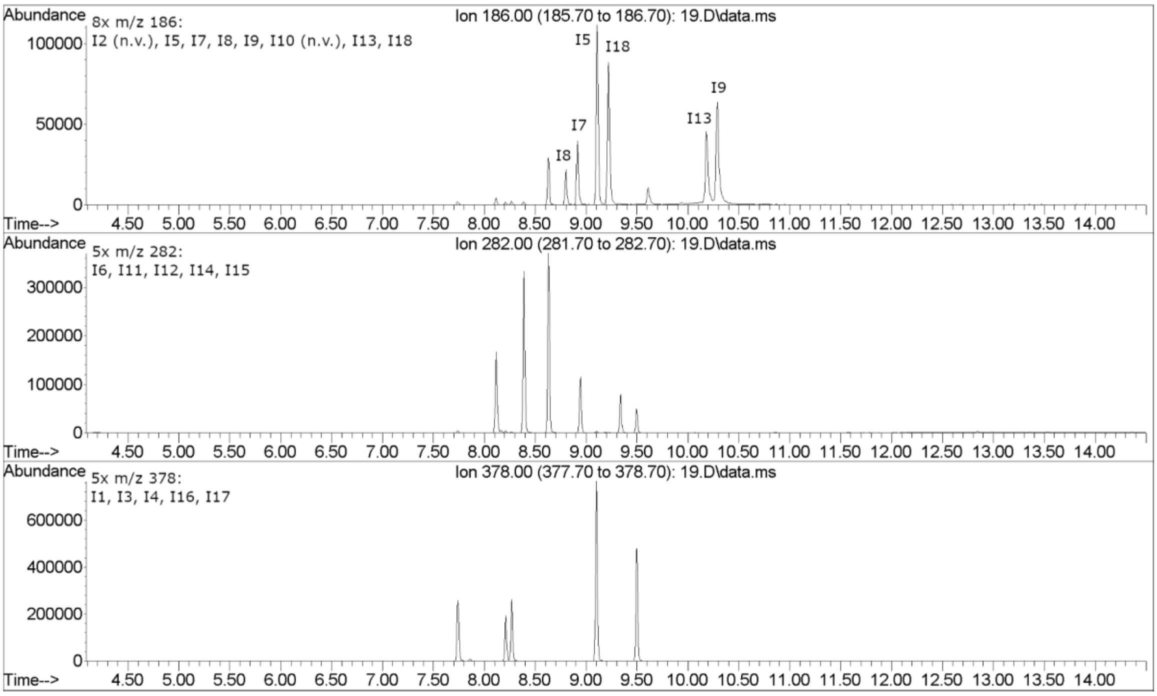

Supplementary Figure S6-2. Extracted ion chromatograms at *m/z* 186 Da, *m/z* 282 Da, and *m/z* 378 Da for the observed TFAA derivatization products of the isomer mixture, n.v. – not visible means that the corresponding isomers cannot be seen in the extracted ion chromatogram due to the low intensity of the peaks (both isomers are acids).

SI7. Validation of the workflow with a test mixture.

Supplementary Table S7. Chemicals included in the test mixture with their corresponding molecular weight (MW), CAS number, and supplier. In addition the detected Kovats retention index (KRI) and corresponding [*m/z*] are given for the derivatization reagents applied according to the workflow.

N°	Chemical	MW	CAS	Supplier	Initial		TMSH		TFAA		DMF-DMA		NH_4OH / TMSH	
		[g/mol]			KRI	[*m/z*]	KRI	[*m/z*]	KRI	[*m/z*]	KRI	[*m/z*]	KRI	[*m/z*]
19	Vanillin	152.14	121-33-5	Alfa Aesar	1232	152	1456	212	1101	248	1477	212	-	-
20	Benzaldehyde	106.12	100-52-7	Merck	398	106	737	152	-	-	735	152	-	-
21	4-Bromphenol	173.02	106-41-2	Aldrich	1029	173	907	187	725	269	903	187	-	-
22	Ethyldecanoate	200.32	110-38-3	Fluka	1202	200	-	-	-	-	-	-	1111	186
23	Di-*n*-propylphthalate	250.29	131-16-8	Alfa Aesar	1751	250	-	-	-	-	-	-	1344	194
24	Benzophenone	182.22	119-61-9	Alfa Aesar	1575	182	1603	196	-	-	-	-	-	-
25	Valerophenone	162.23	1009-14-9	Aldrich	1157	162	1181	176	-	-	-	-	-	-
26	Mecoprop	214.65	93-65-2	HPC	-	-	1491	228	-	-	1468	228	1477	228
27	Naphthaleneacetic acid	186.21	86-87-3	Fluka	1772	186	1711	200	-	-	1697	200	1709	200
28	Phenylbenzoate	198.22	93-99-2	Alfa Aesar	1610	198	-	-	-	-	-	-	709	136

SI8. Retention indices for the selected isomers and their corresponding derivatization products.

Supplementary Table S8. Isomers ([*m/z*] 186 Da) and their corresponding derivatization products with Kovats retention index (KRI) and corresponding [*m/z*] are given for the derivatization reagents applied according to the workflow.

N°	Initial	TMSH		TFAA		DMF-DMA		Borate		NH_4OH	
	KRI	KRI	[m/z]	KRI	[*m/z*]	KRI	[*m/z*]	KRI	[*m/z*]	KRI	[*m/z*]
**1**	1665	1695	214	1290	378	1695	214	1749	242	-	-
**2**	1755	1716	200	1705	378	1719	200	1757	242	-	-
**3**	1897	2062	214	1424	378	1819	214	1751	242	-	-
**4**	2124	2008	214	-	-	2008	214	-	-	-	-
**5**	1618	-	-	-	-	-	-	-	-	1390	172
**6**	1737	1776	214	1670	282	1725	200	1813	195	1625	144
**7**	1570	1365	158	-	-	-	-	-	-	-	-
**8**	1542	1359	158	-	-	-	-	-	-	1310	144
**9**	1893	1936	201	-	-	1933	201			-	-
**10**	1788	1740	200	-	-	1744	200	504	281	-	-
**10**	-	1779	214	-	-	-	-	-	-	-	-
**11**	1745	1668	200	1508	282	1660	200	1832	207	-	-
**12**	1724	1801	214	1582	282	1668	200	-	-	-	-
**12**	-	1801	214	-	-	1590	181	-	-	-	-
**13**	1871	1875	200	-	-	1867	201	1604	275	-	-
**14**	1745	1648	200	1464	282	1641	200	1819	207	-	-
**15**	1508	1570	200	1392	282	1570	200	1574	207	-	-
**16**	1780	-	-	1407	378	1716	214	1821	242	-	-
**17**	2111	1955	214	1620	378	1955	214	1835	242	-	-
**18**	1644	-	-	-	-	-	-	-	-	1414	172

Supplementary References

1 K. Yamauchi, T. Tanabe and M. Kinoshita, Trimethylsulfonium hydroxide: a new methylating agent, *J. Org. Chem.*, 1979, **44**, 638–639.

2 P. Gries, A. S. Rathore, X. Lu, J. Chiou, Y. B. Huynh, A. Lodi and S. Tiziani, Automated Trimethyl Sulfonium Hydroxide Derivatization Method for High-Throughput Fatty Acid Profiling by Gas Chromatography-Mass Spectrometry, *Molecules (Basel, Switzerland)*, 2021, **26**. DOI: 10.3390/molecules26206246.

3 K. P. C. Vollhardt and N. E. Schore, *Organische Chemie. Deluxe Set*, Wiley-VCH, Weinheim, 2020.

4 N. Hashimoti, T. AOYAMA and T. Shioiri, New methods and reagents in organic synthesis. 14. A simple efficient preparation of methyl esters with trimethylsilyldiazomethane (TMSCHN2) and its application to gas chromatographic analysis of fatty acids, *Chem. Pharm. Bull.*, 1981, **29**, 1475–1478.

5 E. Kühnel, D. D. P. Laffan, G. C. Lloyd-Jones, T. Del Martínez Campo, I. R. Shepperson and J. L. Slaughter, Mechanism of methyl esterification of carboxylic acids by trimethylsilyldiazomethane, *Angewandte Chemie (International ed. in English)*, 2007, **46**, 7075–7078.

6 I. Tot, Y. Müller, C. Werner, T. Rosenau and A. Potthast, A novel, mild and selective methylation of carboxyl groups in cellulosic pulps 10th EWLP, Stockholm, Sweden, August 25–28, 2008, *Holzforschung*, 2009, **63**. DOI: 10.1515/HF.2009.103.

7 F. Pippig, S. Sarghini, A. Holländer, S. Paulussen and H. Terryn, TFAA chemical derivatization and XPS. Analysis of OH and NHx polymers, *Surf. Interface Anal.*, 2009, **41**, 421–429.

8 P. Belov, V. L. Campanella, A. W. Smith and R. Priefer, Microwave-assisted methylation of phenols with DMF-DMA, *Tetrahedron Letters*, 2011, **52**, 2776–2779.

9 Y. Chen, Recent Advances in Methylation: A Guide for Selecting Methylation Reagents, *Chemistry (Weinheim an der Bergstrasse, Germany)*, 2019, **25**, 3405–3439.

10 P. Starkov and T. D. Sheppard, Borate esters as convenient reagents for direct amidation of carboxylic acids and transamidation of primary amides, *Organic & biomolecular chemistry*, 2011, **9**, 1320–1323.

11 J. T. Reeves, M. D. Visco, M. A. Marsini, N. Grinberg, C. A. Busacca, A. E. Mattson and C. H. Senanayake, A General Method for Imine Formation Using B(OCH2CF3)3, *Organic letters*, 2015, **17**, 2442–2445.
